# Alpaca. A Simplified and Reproducible Python‐Based Pipeline for Absolute Proteome Quantification Data Mining

**DOI:** 10.1002/pmic.202400417

**Published:** 2025-04-26

**Authors:** Borja Ferrero‐Bordera, Dörte Becher, Sandra Maaß

**Affiliations:** ^1^ Department of Microbial Proteomics Institute of Microbiology Center of Functional Genomics of Microbes University of Greifswald Greifswald Germany; ^2^ Institute of Medical Psychology Medical Faculty Ludwig‐Maximilians‐University Munich Munich Germany

**Keywords:** absolute proteome quantification, data mining, open source, protein abundances, proteomics, proteomics analysis, Python

## Abstract

The accurate construction of computational models in systems biology heavily relies on the availability of quantitative proteomics data, specifically, absolute protein abundances. However, the complex nature of proteomics data analysis necessitates specialised expertise, making the integration of this data into models challenging. Therefore, the development of software tools that ease the analysis of proteomics data and bridge between disciplines is crucial for advancing the field of systems biology. We developed an open access Python‐based software tool available either as downloadable library or as web‐based graphical user interface (GUI). The pipeline simplifies the extraction and calculation of protein abundances from unprocessed proteomics data, accommodating a range of experimental approaches based on label‐free quantification. Our tool was conceived as a versatile and robust pipeline designed to ease and simplify data analysis, thereby improving reproducibility between researchers and institutions. Moreover, the robust modular structure of Alpaca allows its integration with other software tools.

**Abbreviations**
GUI: graphical user interface

1

Systems biology is the field that tries to uncover the complexity behind life in a comprehensive way. This means moving the focus from isolated players of life such as particular genes or proteins and start to understand nature from an integrative outlook as in the case of metabolic or regulatory networks [1]. The final aim of systems biology is to produce computational models capable of recreating and explaining the complexity enclosed in living systems.

Quantitative MS‐based proteomics emerged in the last decade as a powerful tool for inspecting how protein abundance changes in a biological system under different conditions [2, 3]. This technology has become available for many institutions and is meanwhile extensively applied. However, quantitative proteomics is mostly relative, and therewith able to describe changes in protein abundance between conditions. Although this is sufficient for many studies, it does not match the requirements for producing accurate computational models such as resource balance analysis or flux balance analysis.

Preferably, absolute protein abundance data are available to build systems biology models. Nevertheless, this kind of datasets are still scarce. Currently, accessible datasets on the Protein Abundance Database (PaxDb) are limited to a few species across the domains of life, having data only for 67 eukaryotes, 81 bacteria and 22 archaea [4] (on 8 June 2024). Even for model organisms such as *Drosophila melanogaster*, *Caenorhabditis elegans*, or *Bacillus subtilis*, not many datasets are accessible. Hence, the number of available datasets is still far from the requisites to train accurate prediction models.

In recent years, methodological advances have pushed the possibilities of producing absolute proteomics data. Such protocols have eased the sample preparation, maximised the output or allowed to reach challenging protein fractions such as the membrane proteome [5, 6, 7]. However, these technical advances have not turned into a significant output yet.

One reason that might keep the dataset production behind could be the complexity of MS‐based proteomics applications and analysis. This could compromise the accessibility to tailored datasets for many researchers. Considerable field‐bridging efforts have been made with user‐friendly software such as MaxQuant or Perseus [8, 9]. Indeed, in recent years, there is a growing interest in the development of tools like Amica or protti that aim to improve and expand the already available software [10, 11]. However, these tools have mostly focused on relative protein quantification approaches.

Here, we introduce Alpaca (AbsoLute Protein quAntifiCAtion), which is conceived as a Python‐based pipeline to mine high‐value absolute quantification data from unprocessed proteomics datasets. Alpaca makes absolute protein quantification more accessible by significantly reducing and simplifying data analysis and mining. Our tool offers versatile and reproducible data analysis openly accessible for all researchers in the field. Alpaca is capable of analysing large datasets in less than 10 s (details are described in Supporting Information). Previously, only the R‐package aLFQ focused on absolute protein quantification [12]. Alpaca differentiates from this software by its versatility as our software allows to integrate enrichment of particular proteome fractions.

Python was chosen for the development of Alpaca because it is an open‐source programming language that is widely used in the scientific community, especially for analysing large datasets. This choice benefits the community as many tools for training models have been developed in Python. Moreover, Pandas library was used for framing the data, as it is an extended library in data analysis in Python allowing to easily visualise and export the quantified data in commonly used formats such as text or Excel files. Thus, the usage of Python, specifically Pandas, offers the possibility of easily interconnect Alpaca analysis with other available software.

Proteome‐scale absolute quantification is usually performed by regression to log‐transformed intensities of anchor proteins added at known amounts. To do so, Alpaca was designed to identify and process any label‐free quantification method used to generate the data. Commonly used data processing protocols for intensity normalisation and imputation were implemented to improve quantification capabilities.

Moreover, the pipeline was optimised for intact protein standards (e.g., Universal Proteomics Standards) due to their advantages compared to other approaches and its common usage in the community [13]. Nonetheless, Alpaca could be adapted to other protocols, for example, using stable isotope labelled standards or concatemers for absolute quantification.

Alpaca is the first framework to also include a module for enrichments of proteome fractions, a necessary module as certain protocols contain a step in which native concentrations of proteins are modified (e.g., during membrane protein enrichment or extracellular protein concentration).

Alpaca is released in two different formats, as a modular Python package and as a user‐friendly graphical user interface (GUI). The developed pipeline is suitable for quick processing of absolute proteome quantification experiments aimed for computational model approaches. The here described workflow is for Alpaca version 1.0.

Alpaca pipeline is compatible with the protein groups report from some of the most used proteomics engines (MaxQuant, MSFragger, DiaNN and Spectronaut). The software contains four modules: data preprocessing, quantification, sample preparation and data integration (Figure 1). This modular design makes the software simpler, more flexible and robust to different experimental preparations.

**FIGURE 1:**
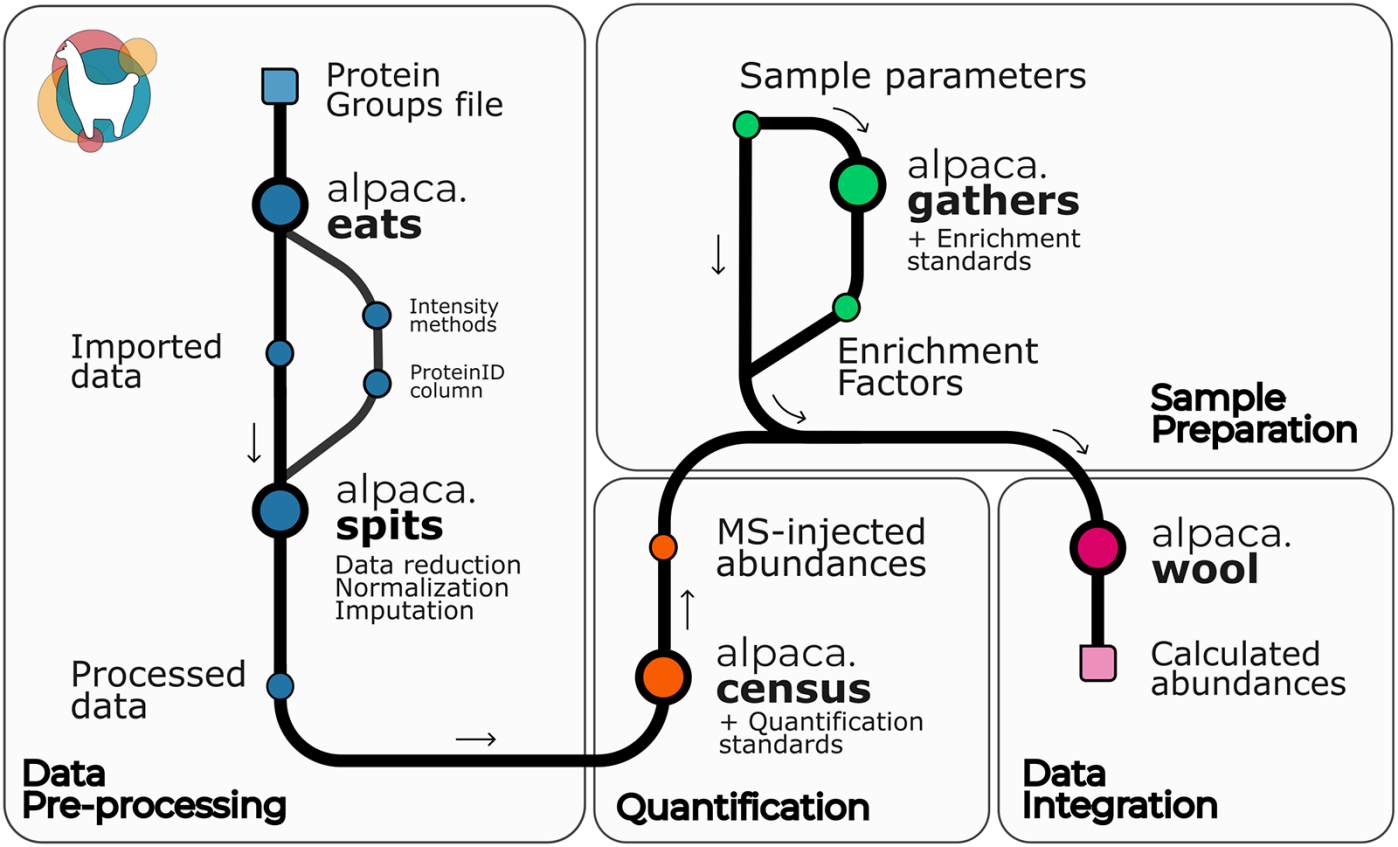
The structure of the data analysis pipeline is shown, describing its main steps and the different input and outputs. Briefly, search engine output (e.g., proteingroups.txt) is processed and formatted using the functions enclosed in the Data preprocessing module, which is followed by a census function for quantification based on protein standards. MS‐measured abundances can be translated to sample abundances by adding experimental details. In case a subproteomic enrichment step is needed, there is a module for sample preparation that integrates experimental details, measured data and information on protein standards to quantify the enrichment. Finally, all information can be integrated using the wool function.

The data preprocessing module is composed by the functions *alpaca.eats* and *alpaca.spits*. The first function, *eats*, imports the data returning a dataframe. Moreover, if the function is set to inspect (by default is True), a dictionary with the different intensity methods identified in the data and a suggested ProteinID column are returned. These outputs can be incorporated into the function *spits*, which can identify sample groups. Grouping accuracy was tested in silico, performing correctly in 97.1% of the tests (*n* = 54,857) (details are described in Supporting Information). This feature reduces the manual steps that the user needs to perform for data analysis. The resulting outputs can be processed through the function *spits*, which incorporates the common steps necessary for proteomics analysis such as contaminant removal, data filtering, missing values imputation and intensity normalisation. Thus, this module can also be applied to speed up routine proteomics data processing.

Quantification is concentrated in one Python function (*census*), which fits the added anchor proteins to the measured intensities. Added anchor protein amounts can be imported as text or Excel files. In case the user has no preference, the function can calculate the most suitable intensity method (this is described further in the pipeline tutorial in Supporting Information). Visualisation of the fitted standards is included as an option. Finally, the function returns the calculated abundances from the MS‐measured sample.

Additionally, the package incorporates a function (*alpaca.Consultant*), which aims to assist the user in the analysis by suggesting the best data processing options for quantification. This function takes the raw ProteinGroups file and the anchor proteins file to assess which intensity and normalisation method fits the data better.

Finally, two more functions, *gathers* and *wool*, were designed to connect the MS data to the experimental details of sample preparation.

Sample preparation information (e.g., sample volumes, protein concentrations and cell numbers) can be added as described in Table 1 and integrated to the calculation through the function *alpaca.wool*. This function transforms the measured MS‐abundances with the experimental details to yield high‐valuable absolute quantification data (total fmol, ng/µg of protein and molecules per cell) for systems biology.

**TABLE 1:** Experimental parameters table.

Condition^a^	SampleVolume^b^	ProteinConcentration^c^	AmountMS^d^	CellsPerML^e^	TotalCultureVolume^f^	ProteinSRM^g^	fmolSRM^h^	Enrichment^i^	EnrichmentMode^j^	StdDilution^k^	StdVolume^l^
Cond1_t0	2.31	2.99	9.67	4.54	7.54	TNAMLN	4.44	FALSE			
Cond2_t1	2.5	0.2	4.1	5.13	2.62	AJFVYC	4.85	TRUE	Concentration	10	10
Cond3_t2	7.38	6.56	2.77	3.66	3.8	BYEKSC	9.71	TRUE	Enrichment	2	10

*Note*: This example covers all possible columns. Nonetheless, not all columns are necessary. For example, enrichment columns (EnrichmentMode, StdDilution and StdVolume) are only used if any enrichment step was performed (TRUE). More information about this is described in the section on proteome fraction enrichment in the Supporting Information.

^a^ Condition: Identifier for the condition or timepoint in which the parameters were applied.

^b^ SampleVolume: Volume (in µL) of the protein extract used for digestion.

^c^ ProteinConcentration: Determined protein concentration (µg/µL) in the sample.

^d^ AmountMS: Sample amount (in µg) injected into the mass spectrometer.

^e^ CellsPerML: Determined number of cells per mL in the original culture.

^f^ TotalCultureVolume: Total cultivation volume harvested (in µL).

^g^ ProteinSRM (Optional): If the enrichment of a subcellular fraction has been calculated using targeted proteomics (SRM). This column lists the accession of measured proteins in SRM to calculate the enrichment. If more than one protein has been used, the accessions should be listed with a comma as separator.

^h^ fmolSRM (Optional): If the enrichment of a subcellular fraction has been calculated using targeted proteomics (SRM). Femtomoles of the native target proteins determined by SRM. If more than one protein has been used, the amounts should be listed with a comma as separator.

^i^ Enrichment (Optional): Boolean (True or False). Samples that have been enriched should be specified as true.

^j^ EnrichmentMode (Optional): Type of enrichment–either enrichment or concentration (see Supporting Information).

^k^ StdDilution (Optional): This parameter specifies the dilution of the stock solution of enrichment standards before adding it to the sample. For example, in the table, a 1:10 dilution would correspond to Cond2_t1, and a dilution 1:2 for Cond3_t2. If the standards were not diluted before addition, specify 1. Only used when the enrichment is calculated through the alpaca.gathers() function, then details of the preparation of the used proteins should be specified.

^l^ StdVolume (Optional): Volume of enrichment standards (µL) added to the sample. Only used when the enrichment is calculated through the alpaca.gathers() function, then details of the preparation of the used proteins should be specified.

In case the sample preparation focused on a fraction of the proteome, the function *gathers* allow to quantify and incorporate the enrichment step. This could be the case for membrane or extracellular proteome samples which need further analysis as the native protein amounts are usually modified in an enrichment or concentration step during sample preparation [6, 14]. To quantify this step, *alpaca.gathers* function calculates the enrichment by integrating quantified protein data resulting from the *census* function, together with the experimental details from the experimental parameters table (Table 1) and a set of enrichment standards imported by the user. Finally, the function *gathers* updates the sample preparation parameters with the calculated enrichment factors. Subsequently, when sample preparation is integrated through the *wool* function, abundances are corrected with enrichment factors.

Of note, Alpaca is not capable of directly calculating enrichment for protocols that rely on targeted MS. That could be the case for protocols such as the one described by Antelo‐Varela et al. [6]. The required calculations can be easily done in Skyline software and subsequently added to Alpaca through the experimental parameters table (Table 1).

Besides the Python package that can be used and integrated with other coding libraries, a GUI was developed which is freely accessible at https://alpaca.nube.uni‐greifswald.de/. Alpaca GUI is as powerful and simple as the Python package but wrapped in a user‐friendly environment.

The GUI structure was designed to make analysis intuitive without compromising the capabilities of the coding version. The display is divided into two main sections: the sidebar, which controls data input and user options for the analysis (data reduction, normalisation, imputation and quantification standards); and the GUI body, where experimental settings (sample preparation and enrichment standards) and data are visualised.

To strengthen the analysis capabilities with the GUI, a visualisation module was added. The GUI visualisation module provides the possibility to assess data quality and have insights on the quantification without the need of coding skills (Figure 2).

**FIGURE 2:**
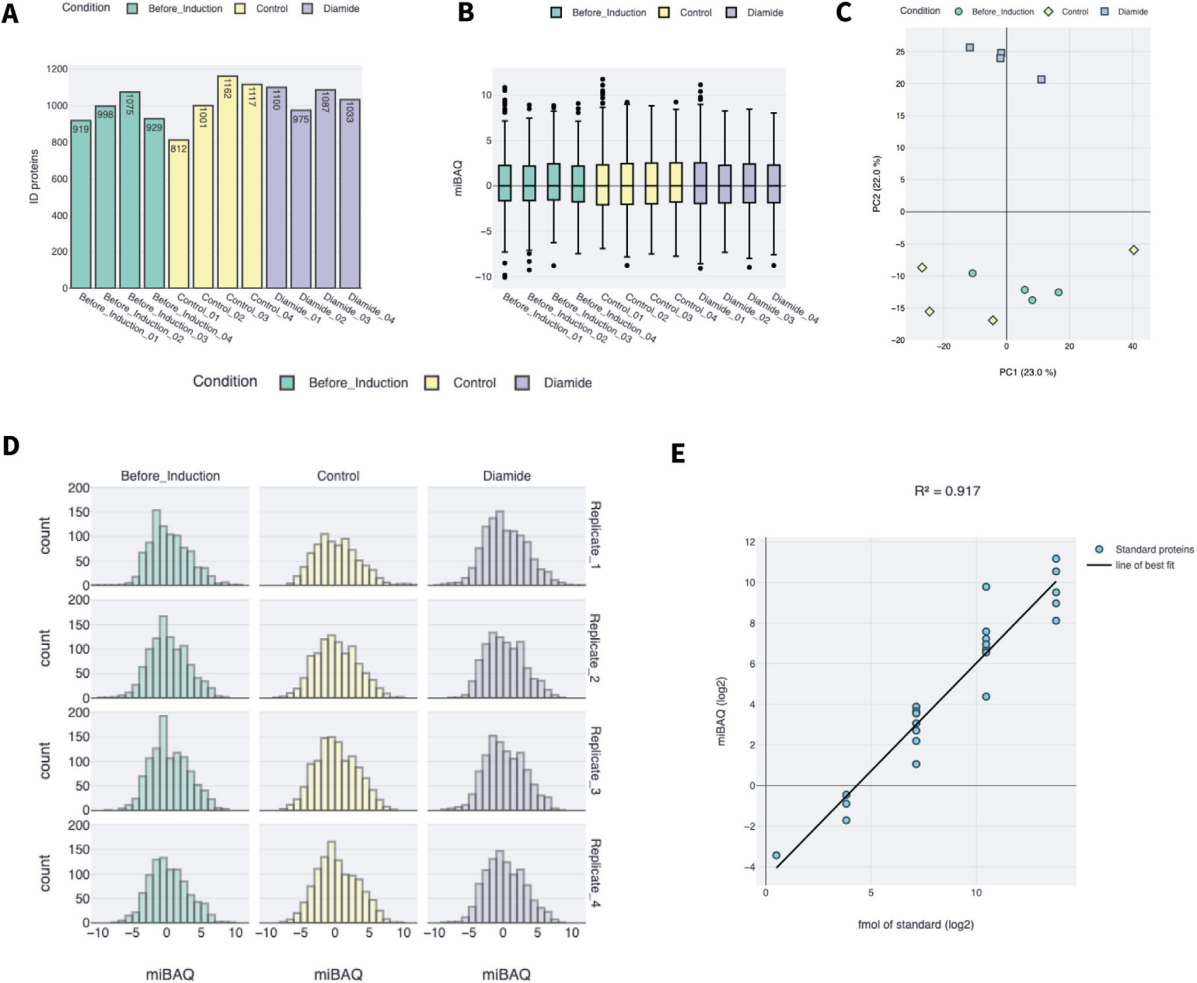
Example of the visualisation capabilities integrated in the graphical user interface (GUI) using the dataset published in Ferrero‐Bordera et al. (2024) [14] as example data. (A) Number of quantified proteins per sample which can be visualised through the tab quantified proteins. (B) The intensity boxplot can be inspected on intensities tab. (C) Principal component analysis (PCA) can be plotted on PCA tab. (D) Distribution plot for each sample can be visualised on the distribution plot tab. (E) The calibration curve with the anchor proteins used for absolute quantification of the proteome is plotted on the calibration curve tab together with the *R* ^2^.

In this article, Alpaca, a Python‐based pipeline, is introduced. This pipeline is freely available as a modular Python package and implemented into a web‐based GUI. Alpaca is designed to simplify absolute protein abundances data mining for computational modelling. Our pipeline simplifies proteomics data analysis with a versatile, and robust modular structure. We believe that the development of such tools in a time in which large datasets are a powerful resource is key for the development of systems biology. Thus, these tools streamline data processing and analyses improving reproducibility between different labs.

A pipeline tutorial as well as links to extended documentation and a video tutorial can be found in the Supporting Information.

## Author Contributions

B.F.B. conceptualised, designed and created the Python Library and the GUI. All authors read and approved the final manuscript. D.B. and S.M. substantially revised the pipeline and contributed to the manuscript writing. All authors read and approved the final manuscript.

## Conflicts of Interest

The authors declare no conflicts of interest.

## Supporting information



Supporting Information

## Data Availability

Data sharing not applicable to this article as no datasets were generated or analysed during the current study.
