# Supplementary material for: Alpaca. A Simplified and Reproducible Python‐Based Pipeline for Absolute Proteome Quantification Data Mining
Source: Proteomics. 2025 Apr 26;25(9-10):e202400417. doi: 10.1002/pmic.202400417 (PMC12076531; doi:10.1002/pmic.202400417)
Supplement: Supplementary file 1 — Supporting Information [file PMIC-25-e202400417-s001.docx]

**Supporting Information**

Alpaca. A simplified and reproducible Python-based pipeline for absolute proteome quantification data mining

Borja Ferrero-Bordera^1^, Dörte Becher^1^, and Sandra Maaß^1#^

^1^ Department of Microbial Proteomics, Institute of Microbiology, Center of Functional Genomics of Microbes, University of Greifswald, 17489 Greifswald, Germany

^#^ **Correspondence**:

Sandra Maaß

University of Greifswald, Institute of Microbiology, Felix-Hausdorff-Str. 8, 17489 Greifswald;

sandra.maass@uni-greifswald.de

Tel.: +49 (0)3834 420 5921

Fax: +49 (0)3834 420 5902

**Abbreviations**: GUI - graphical user interface;

**Keywords:**  Absolute Proteome Quantification, Protein Abundances, Proteomics, Proteomics Analysis, Data Mining, Python, Open Source

### Testing of Grouping accuracy

**Methods**

**Benchmarking of the pipeline execution time**

To evaluate the execution time of the pipeline, we generated test datasets with varying sizes, ranging from 2 to 9 experimental conditions and from 1,522 to 30,003 unique proteins. For each dataset size, 10 randomized versions were created to vary the values associated with the proteins, and each was subsequently tested. The performance was assessed for both the full protocol, including enrichment quantification, and a simplified version without enrichment. The benchmarking was performed on a MacBook Pro (M1, 2020, 8GB RAM) running macOS 15.3.2.

**Accuracy of condition identification**

To test the accuracy of the functions involved in grouping samples, we selected a set of common word categories used as identifiers (see Supplemental Table 1). Each category included at least 3 different identifiers. We permutated identifiers from up to 4 categories to create all possible combinations among the common identifiers (e.g., "Control_B_1E5_Plasma").

Supplemental Table 1. Common word categories considered in our simulation to generate sample names. Up to 4 categories were combined to generate sample names.

| **ID group** | **List of IDs** |
| --- | --- |
| **Condition** | ['Treatment', 'Control', 'deltaX', 'deltaY', 'GFP', 'Condition', 'KatA'] |
| **Letter** | ['A', 'B', 'C', ''] |
| **Instrument** | ['QExactive', 'Orbitrap', 'Fusion', 'TimsTof', ''] |
| **Sorting** | ['FACS', 'sorted', 'FACSsort', ''] |
| **Cell Number** | ['1E6', '5E6', '5E5', ''] |
| **Digestion Protocol** | ['FASP', 'STrap', 'InSol', 'InGel', 'LiMPS', ''] |
| **Project ID** | ['PR001', 'PR010', 'PR002', 'PR012', ''] |
| **Sample Type** | ['Plasma', 'Tissue', 'Supernatant', 'Nuclei', ''] |

Next, we generated different test sizes ranging from 5 to 100 different sample names (e.g., ["Control_B_1E5_Plasma", "Control_B_1E5_Tissue", "Control_B_1E5_Supernatant", "Control_B_1E6_Plasma", "Control_B_5E6_Plasma"]). For each generated sample name, 5 replicates were created by adding the replicate number as a suffix (e.g., "_1") and "Intensity" as a prefix to mimic common intensity nomenclature.

Finally, we tested the accuracy of the grouping functions. The accuracy was determined by calculating the percentage of correctly grouped identifiers out of the total number of identifiers. The code is available on <https://github.com/borfebor/alpaca_proteomics/blob/main/Test/Accuracy_test.ipynb>

**Results**

**Benchmarking of the pipeline execution time**

As shown in the Supplemental Figure 1, execution time increased with dataset size for both tested protocols (see Supplementary Mehods). Datasets with a larger number of experimental conditions generally required more time to process. The protocol including enrichment was slightly more computationally intensive, with execution times reaching up to ~6.5 seconds for the largest datasets (30,000 proteins and 9 conditions). In contrast, the protocol without enrichment showed slightly faster performance, with maximum execution times around ~5.5 seconds for similarly sized datasets.

Overall, the pipeline maintained fast execution across all tested conditions. Even for the most complex datasets, the runtime remained under 7 seconds, highlighting the efficiency and scalability of the implementatio

**
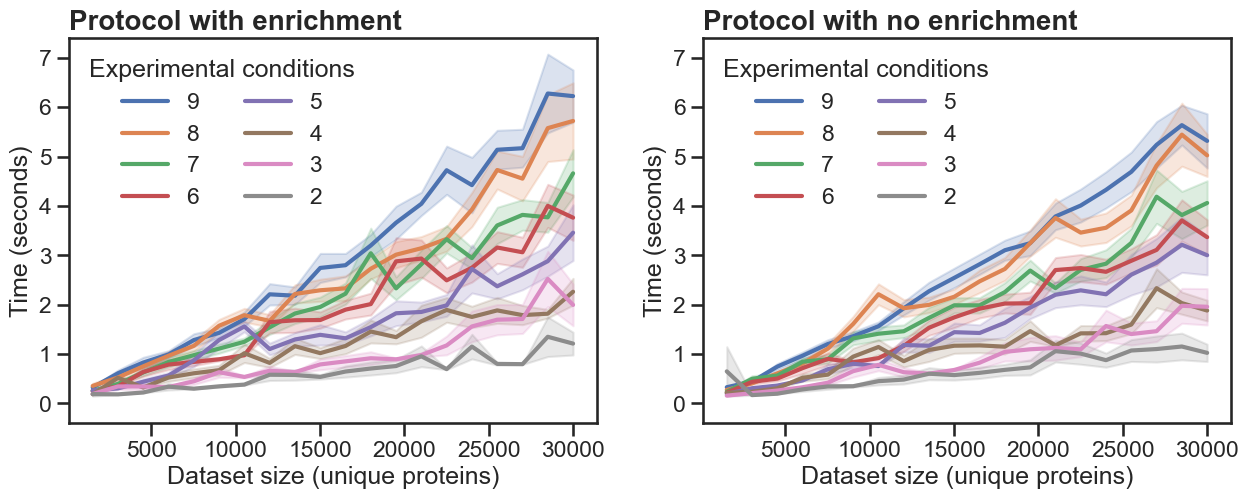
**

Supplemental Figure 1. Execution times (in seconds) were measured for datasets ranging from 1,522 to 30,003 unique proteins and from 2 to 9 experimental conditions. The left panel shows results for the protocol including enrichment quantification, while the right panel shows results for the simplified protocol without enrichment. Each line represents the mean execution time across 10 randomized datasets for each condition, with shaded areas indicating standard deviation. Execution was performed on a MacBook Pro (M1, 2020, 8 GB RAM) running macOS 15.3.2.

**Accuracy of condition identification**

Generated samples were grouped with an average accuracy of 97.1%. Grouping performed specially well on simulated experiments with less than 20 samples, in which the lowest average accuracy was 99.1% when 3 different identifiers were combined (Supplemental Figure 2A). Lower accuracies were observed with the increase of sample names per test. The lowest average accuracy was 86.5% when testing sets between 70 to 80 groups with 4 chained identifiers. An decrease on accuracy was observed by the addition of identifiers to the group names, showing that groups containing 4 identifiers where classified less accurately than groups with 3 identifiers. The decrease on accuracy ocurred mainly when grouping samples with longer names in which the last identifier was from the word categories “Cell Number” or “Project ID” (Supplemental Figure 2B). This was probably due to the problems of the algorithm to classify changes of 1 digit in longer group names.


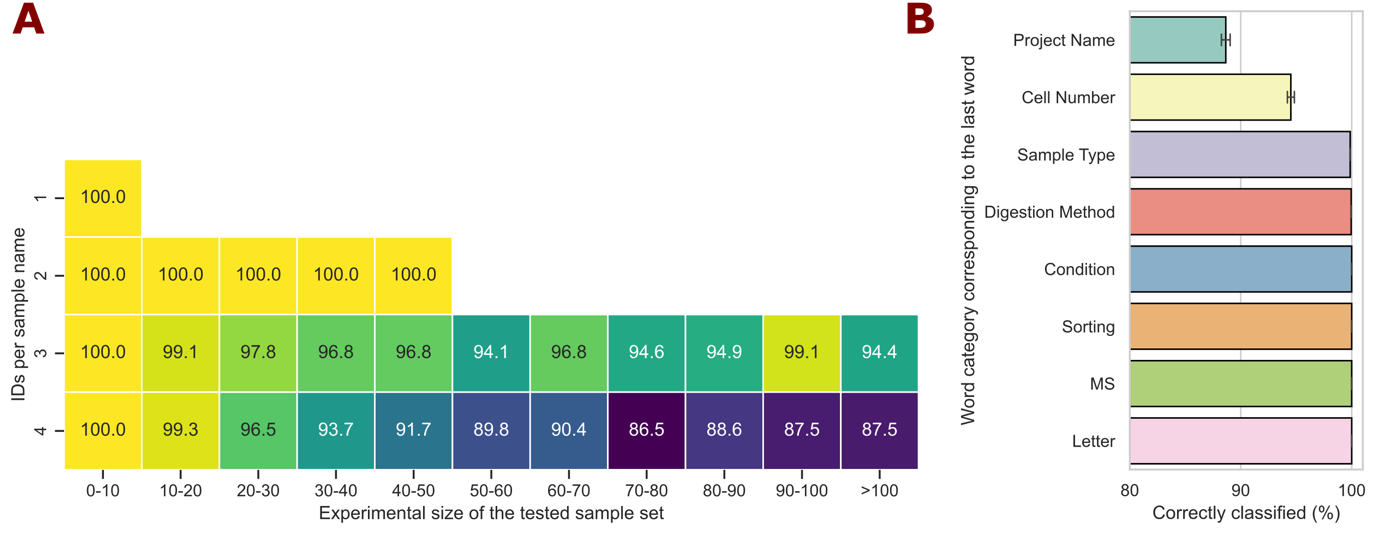


Supplemental Figure 2. A) Average classification accuracy of sample groups based on the amount of words from a given category (Supplemental Table 1) combined in the sample name (Y axis) and the number of samples contained with 5 replicates contained in the simulated experiment (X axis). B) Average percentage of correctly grouped samples based on the last word of the generated sample names.

# Pipeline Tutorial

Extended documentation on the different functions and a user-friendly tutorial can be found in the documentation page (<https://borfebor.github.io/alpaca_proteomics/>). Additionally, the tutorial can be found as a Python notebook in the following link (<https://github.com/borfebor/alpaca_proteomics/blob/main/Tutorial/Tutorial.ipynb>). A tutorial video can be found at <https://grypstube.uni-greifswald.de/w/xrmy6G55kbrjd3BFcLLSfG>.

First step

1. Install alpaca package on the terminal through

pip install alpaca_proteomics

2. Import the package

1. from alpaca_proteomics import alpaca

Data import and formatting

Alpaca works with unprocessed proteomics datasets (in this example`proteinGroups.txt` from MaxQuant). The package takes the file which can be found in the combined folder of MaxQuant output.

2. file = 'proteinGroups.txt'

Data importation

3. df, id_col, intensity_dict = alpaca.eats('proteinGroups.txt')

The function returned:

- *df* is the imported data as a pandas dataframe
- *id_col* corresponds to the column which was detected to contain the Protein IDs
- *intensity_dict* is a dictionary which groups the columns containing intensity data within each intensity method (e.g. LFQ)

In our example, the data contained 3 intensity methods (Intensity, iBAQ, LFQ)

Assistance on the analysis (Optional)

In case the user is unsure on which parameters are the most optimal to process the data, the package incorporates a function (*Consultant*) which can quickly explore and suggest the best parameters for the analysis. The function returns a table with the calculated fitting score for each intensity method available in the data and its respective normalization.

1. # Path to the anchor proteins file

2. standards_file = 'UPS2.xlsx'

3.

4. # Importation of the anchor proteins file (more details on these are listed below)

5. st_proteins = alpaca.eats(standards_file)

6.

7. # Samples in which anchor proteins were added

8. spiked_samples = ['Control_01', 'TreatmentA_01', 'TreatmentB_01']

9.

10. # Valid values per condition

11. values_per_sample = 1/4

12.

13. suggested = alpaca.Consultant(df,

14. st_proteins,

15. intensity_dict,

16. added_samples=spiked_samples,

17. values_per_sample=values_per_sample)

18.

Data pre-processing

﻿This module processes a DataFrame for quantitative analysis, performing tasks such as cleaning, transformation, imputation, normalization, and reformatting based on provided parameters.

1. # Data pre-processing

2.

3. values_per_sample = 1/4

4.

5. clean_df = alpaca.spits(df,

6. lfq_method='iBAQ',

7. formatting=True,

8. valid_values=values_per_sample,

9. normalization='Median',

10. info_cols=['Accession', 'Gene names'])

Anchor protein quantification

Absolute quantification using Alpaca is optimised for label-free methods, relying on the addition of a set of anchor proteins at a know amount (Supplemental Table 2).

Supplemental Table 2. Format for the file describing the stock solution of anchor proteins.

| Accession | MW (kDa) | Amount (fmol) |
| --- | --- | --- |
| P02768 | 10.1 | 50 |
| Q9Y6K9 | 65.8 | 100 |
| P05067 | 32.5 | 25 |
| O75475 | 48.2 | 75 |
| Q00653 | 20.9 | 30 |

1. # Import the file containing the information about the quantification standards proteins

2.

3. standards_file = 'UPS2.xlsx'

4. st_proteins = alpaca.eats(standards_file)

5.

6. # If applicable, define which samples/replicates contain standards proteins

7.

8. spiked_samples = ['iBAQ Control_01', 'iBAQ TreatmentA_01', 'iBAQ TreatmentB_01']

9.

10. # Quantify the fmol present in the measured samples

11.

12. quant_df, st_proteins, coef, inter, r2 = alpaca.census(clean_df,

st_proteins,

lfq_col='iBAQ',

filter_col = 'Sample',

added_samples = spiked_samples)

The function alpaca.census() adds a column to the processed data with the calculated mol amounts present in the measured samples.

Experimental details

Sample preparation can be connected to the quantified numbers by importing the experimental details as detailed in the main text, Table 2.

1. experiment = “experimentalDetails.csv”

2.   sample_prep = alpaca.eats(experiment)

Experimental details (in our example “experimentalDetails.csv”) can be added as txt, csv or xlsx formats.

Proteome fraction enrichment (Optional)

In case the study focuses in a fraction of the proteome (e.g., membrane proteome or exoproteome), it is likely that during the sample preparation and enrichment step was performed. This module allows to translate the enrichment step to the data based on how the samples were prepared.

Enrichment factors (ER) are calculated based on the fmol quantified in the enriched sample to the raw or non-enriched sample:

$$ER=\frac{{fmol}_{enriched}}{{fmol}_{not enriched}}$$

For that purpose, there are two strategies that are currently covered under our pipeline:

1. **The quantification of specific proteins of the analysed fraction on both before and after the enrichment step using Targeted MS (SRM).**

This strategy was described on Antelo-Varela et al. [1] and relies on using external softwares (e.g., Skyline) to quantify the enrichment step. Enrichment factors can be added to the parameters table under the column “EnrichmentFactor”. Additionally, the SRM quantified amount for a given protein can be added on the columns `ProteinSRM` (accession of the quantified protein) and `fmolSRM` (quantified fmol in the analysed proteome fraction).

1. **The addition of standard proteins at known concentration before performing the enrichment step.**

This approach was described on Ferrero-Bordera et al. [2] and requires a protein mixture at known concentration added before the enrichment step. Used standards have to be formatted as specified in Supplemental Table 3.

Supplemental Table 3. Format for the file describing the applied enrichment standards

| Accession | MW (kDa) | StdConcentration (µg/µl) |
| --- | --- | --- |
| P02768 | 10.1 | 2.5 |
| Q9Y6K9 | 65.8 | 0.8 |
| P05067 | 32.5 | 1.2 |
| O75475 | 48.2 | 3 |
| Q00653 | 20.9 | 2 |

Additionally, the parameters table should contain the Enrichment columns (Enrichment, EnrichmentMode, StdDilution, StdVolume).

- **Enrichment** (True or False): True for those samples that have been enriched.

- **EnrichmentMode** (“Enrichment” or “Concentration”): “Enrichment” corresponds to samples in which the amounts of the target proteins after the enrichment are higher compared to the original proteome (e.g., Membrane proteome). “Concentration” applies to samples in which a fraction of the total target proteome is concentrated to facilitate sample preparation (e.g., exoproteome).

- **StdDilution**: Dilution factor of the enrichment standard stock solution prior adding it to the samples. In case the standards where not diluted, the value is 1.

- **StdVolume**: Added volume (µL) into the sample before the enrichment step.

1. standards_file = 'standards_file.txt'

2. enrichment_std = alpaca.eats(standards_file)

Detailed in Supplemental Table 3. Accepts .txt, .csv and .xlxs

1. sample_prep_updated = alpaca.gathers(quant_df, enrichment_std, sample_prep)

Data integration

This module connects the protein amounts quantified in the sample and the sample preparation, thus allowing to calculate protein amounts to the original state (e.g. bacterial culture, raw culture supernatant). This step yields absolute abundance data (e.g., molecules per cell, fmol /µmol of protein extract)

1. results = alpaca.wool(quant_df, sample_prep_updated)

# References

[1] Antelo-Varela M, Bartel J, Quesada-Ganuza A, Appel K, Bernal-Cabas M, Sura T, et al. Ariadne’s thread in the analytical labyrinth of membrane proteins: Integration of targeted and shotgun proteomics for global absolute quantification of membrane proteins. Anal Chem 2019;91:11972–80. https://doi.org/10.1021/acs.analchem.9b02869.

[2] Ferrero-Bordera B, Bartel J, van Dijl JM, Becher D, Maaß S. From the outer space to the inner cell: deconvoluting the complexity of *Bacillus subtilis* disulfide stress responses by redox state and absolute abundance quantification of extracellular, membrane, and cytosolic proteins. Microbiol Spectr 2024. https://doi.org/10.1128/spectrum.02616-23.
